# The Biomechanical Characteristics of the Strongman Yoke Walk

**DOI:** 10.3389/fspor.2021.670297

**Published:** 2021-04-26

**Authors:** Benjamin R. Hindle, Anna Lorimer, Paul Winwood, Daniel Brimm, Justin W. L. Keogh

**Affiliations:** ^1^Faculty of Health Sciences and Medicine, Bond University, Gold Coast, QLD, Australia; ^2^Sports Performance Research Institute New Zealand, Auckland University of Technology Millennium Institute, Auckland University of Technology University, Auckland, New Zealand; ^3^Faculty of Health, Education and Environment, Toi Ohomai Institute of Technology, Tauranga, New Zealand; ^4^Faculty of Medicine, University of Queensland, Herston, QLD, Australia; ^5^Cluster for Health Improvement, Faculty of Science, Health, Education, and Engineering, University of Sunshine Coast, Sunshine Coast, QLD, Australia; ^6^Kasturba Medical College, Manipal Academy of Higher Education, Manipal, India

**Keywords:** biomechanics, kinematics, spatiotemporal, motion capture, strongman, powerlifting, weightlifting

## Abstract

The yoke walk is a popular strongman exercise where athletes carry a heavily loaded frame balanced across the back of their shoulders over a set distance as quickly as possible. The aim of this study was to use ecologically realistic training loads and carry distances to (1) establish the preliminary biomechanical characteristics of the yoke walk; (2) identify any biomechanical differences between male and female athletes performing the yoke walk; and (3) determine spatiotemporal and kinematic differences between stages (intervals) of the yoke walk. Kinematic and spatiotemporal measures of hip and knee joint angle, and mean velocity, stride length, stride rate and stance duration of each 5 m interval were taken whilst 19 strongman athletes performed three sets of a 20 m yoke walk at 85% of their pre-determined 20 m yoke walk one repetition maximum. The yoke walk was characterised by flexion of the hip and slight to neutral flexion of the knee at heel strike, slight to neutral extension of the hip and flexion of the knee at toe-off and moderate hip and knee range of motion (ROM), with high stride rate and stance duration, and short stride length. Between-interval comparisons revealed increased stride length, stride rate and lower limb ROM, and decreased stance duration at greater velocity. Although no main between-sex differences were observed, two-way interactions revealed female athletes exhibited greater knee extension at toe-off and reduced hip ROM during the initial (0–5 m) when compared with the final three intervals (5–20 m), and covered a greater distance before reaching maximal normalised stride length than males. The findings from this study may better inform strongman coaches, athletes and strength and conditioning coaches with the biomechanical knowledge to: provide athletes with recommendation on how to perform the yoke walk based on the technique used by experienced strongman athletes; better prescribe exercises to target training adaptations required for improved yoke walk performance; and better coach the yoke walk as a training tool for non-strongman athletes.

## Introduction

Strongman is a competitive strength-based sport which now caters to both male and female athletes of varying age, body mass and physical ability. Strongman exercises are often derived from traditional tests of strength and involve more awkward variations of weightlifting/powerlifting exercises. Such exercises include variations of the squat, deadlift and clean and jerk and heavier versions of common everyday activities such as loaded carries (Harris et al., 2016). While strongman exercises vary across competitions, the most common exercises often require athletes to: lift stones, axles, kegs, sandbags or oversized dumbbells for maximal load or as a set of incremental loads in the shortest time; pull heavy vehicles or flip large vehicle tyres over a distance in the shortest time; or carry loaded frames, kegs or sandbags from one location to another in the shortest time (Keogh and Winwood, 2017).

**Figure 1:**
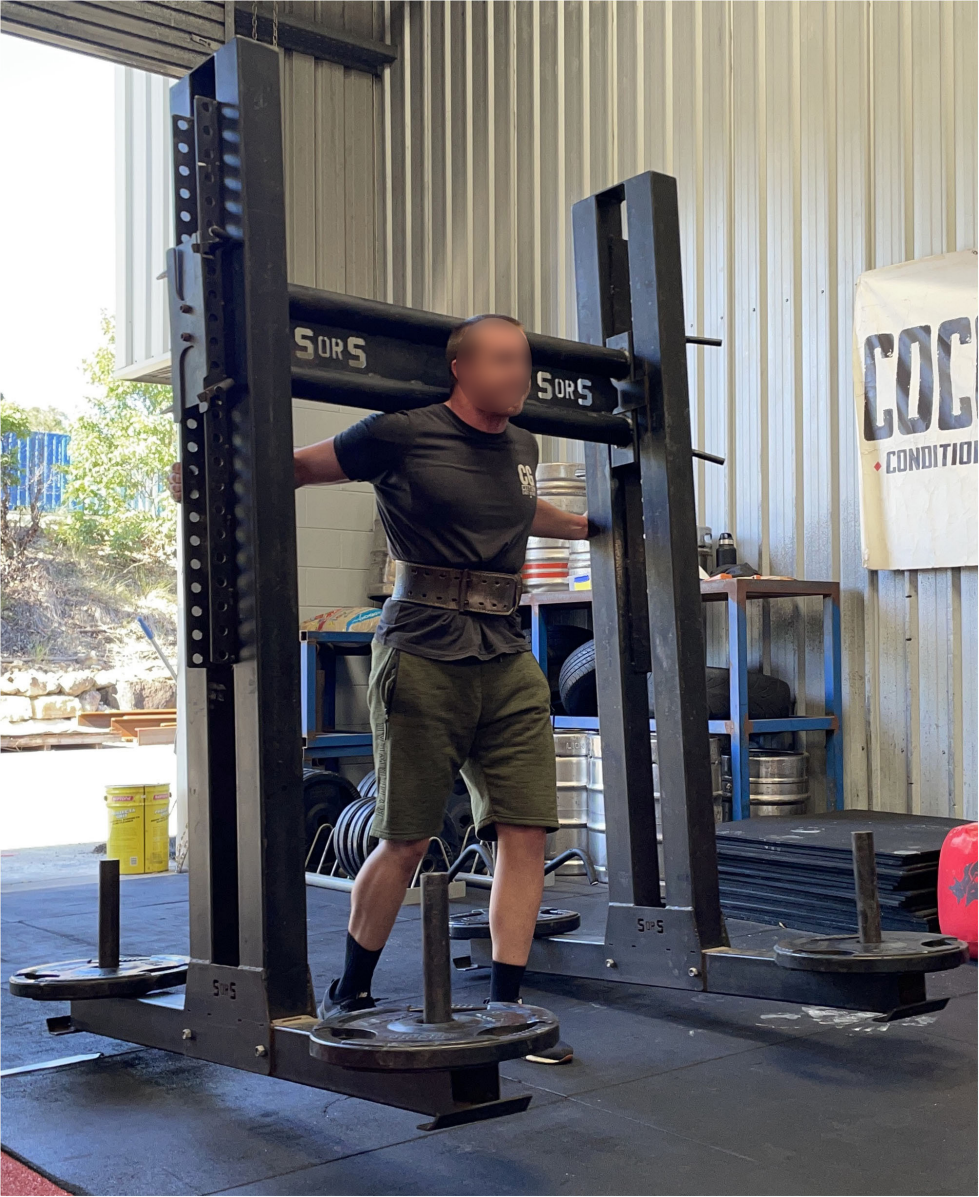
A strongman athlete performing the yoke walk.

The strongman yoke walk requires an athlete to carry a heavily loaded frame balanced across the back of the shoulders a set distance, often 20 m (Figure 1). In strongman training and competition, the yoke walk is typically the heaviest load carriage exercise performed by athletes. The winner of events like the yoke walk, in a competition setting, is the athlete who requires the shortest time to complete the set distance. For those athletes unable to complete the set distance, the distance the yoke was moved from the original starting position is the performance measure.

Research on the biomechanics of the yoke walk is limited. McGill et al. (2009) measured trunk muscle activation patterns and lumbar spine motion, load and stiffness of three experienced male strongman athletes (body mass: 117.3 ± 27.5 kg) performing a single 8 m yoke walk loaded at 177.3 ± 24.3 kg. The large spinal compression observed in athletes performing the yoke walk was suggested to be the result of the greater absolute load of the yoke (when compared with all other implements used in the study including the farmers walk, log lift, tyre flip and atlas stone lift) and the large torso muscular co-contraction required to produce spinal stability throughout the walk (McGill et al., 2009). Beyond the limitation of only including three participants in their study, the loads and carry distance used in the study by McGill et al. (2009) would be considered quite easy by today's standards, whereby athletes of this body mass may be expected to carry loads in excess of 300 kg for at least twice the distance (e.g., 15–20 m) in competition.

A retrospective injury study conducted by Winwood et al. (2014c) revealed 8% of injuries in strongman athletes were caused by the yoke walk, with the most common site of injury during the yoke walk being the lower back. Such findings identified the yoke walk as the second most dangerous strongman exercise with respect to injury causation out of the most popular strongman exercises, with the most dangerous being the atlas stone lift (Winwood et al., 2014c). The greater loads routinely carried by athletes in yoke walk training and competition than in the previous yoke walk study by McGill et al. (2009), coupled with the retrospective data by Winwood et al. (2014c), suggest that athletes are likely exposed to even greater spinal muscular compression and thus greater injury risk than first anticipated by McGill et al. (2009).

Due to the lack of quantitative data on the yoke walk, the biomechanics of loaded backpack carriage and the strongman farmers walk exercise, where competitors are required to carry a heavy object (similar to a suitcase) in each hand, may provide some insight into the likely biomechanics of the yoke walk (Hindle et al., 2019). Differences in lower limb joint kinematics at heel strike and toe off and joint range of motion (ROM) measures have been observed between the farmers walk and unloaded walk (Winwood et al., 2014a). As the farmers walk was characterised by greater flexion of each lower limb joint at heel strike, when compared with unloaded walking, it was concluded that the adopted strategy may reduce braking forces and put the muscle in a better position for force development (Winwood et al., 2014a). Both the farmers walk and backpack load carriage have been associated with an increase in stride rate and a decrease in stride length when compared with unloaded walking, with larger effect sizes reported at greater loads (Winwood et al., 2014a; Liew et al., 2016).

No data exists comparing the biomechanics of male and females performing the yoke walk or in carrying loads similar to those commonly carried in the yoke walk. Biomechanical differences between male and females carrying sub-body mass loads have been reported. When walking at the same velocity (~1.78 m/s) and carrying the same absolute load (≤ ~36 kg distributed as various sites on the body including a rucksack), females exhibited greater forward inclination of the trunk and employed greater stride rate to compensate for their shorter stride length than males (Martin and Nelson, 1986). Martin and Nelson (1986) concluded that females were more sensitive to load than males, with biomechanical differences suggested to be due to the differences in anthropometrics between sexes. Bode et al. (2021) also reported between-sex differences where male soldiers were found to exhibit greater knee ROM than female soldiers when carrying the equivalent absolute vest-borne load (≤ 55 kg) at a set velocity (1.34 m/s). Conversely, Silder et al. (2013) and Krupenevich et al. (2015) found no biomechanical differences between male and females undertaking sub-body mass load carriage, with Krupenevich et al. (2015) concluding that insufficient loading (22 kg) may have accounted for the lack of significant between-sex biomechanical differences. Where Martin and Nelson (1986), Bode et al. (2021), and Krupenevich et al. (2015) used identical absolute loads for male and female participants, Silder et al. (2013) used loads based on a percentage of the carrier's body mass, which may be more representative of the differences in loads used by male and female athletes performing the yoke walk.

The initial acceleration phase (0–3 m) of the farmers walk has been associated with reduced stride lengths, stride rates and increased stance duration and smaller thigh and knee ROM, when compared with the later stages of the walk (8.5–20 m) (Keogh et al., 2014). Due to the lack of between-sex biomechanical analyses performed for heavy load carriage exercises such as the farmers walk or yoke walk, and the inconclusive biomechanical differences between male and female athletes carrying sub-body mass loads (Martin and Nelson, 1986; Silder et al., 2013; Krupenevich et al., 2015; Bode et al., 2021), it is unknown if any two-way interactions exist between sex and interval during heavy load carriage.

As this study is the first of its kind to estimate spatiotemporal and kinematic measures of male and female athletes performing the yoke walk, an emphasis is placed on the importance of undertaking a descriptive-type study of the movement pattern associated with the yoke walk. The aim of this study is to use ecologically realistic training loads and carry distances to: (1) establish the preliminary biomechanical characteristics of the yoke walk; (2) identify any biomechanical differences between male and female athletes performing the yoke walk; and (3) determine spatiotemporal and kinematic differences between stages (intervals) of the yoke walk. In alignment with the aim of this study, it was hypothesised that: (1) athletes performing the yoke walk would exhibit reduced lower limb ROM, stride length and stance duration and increased stride rate when compared with data of the previously studied farmers walk; (2) no between-sex differences would be observed; and (3) athletes would exhibit smaller joint ROM, smaller stride length, reduced stride rate and greater stance duration during the initial 5 m than the later intervals.

By addressing the aim of this study, researchers, strongman coaches and strength and conditioning coaches will be better equipped with an understanding of the yoke walk biomechanics required to: provide male and female strongman athletes with recommendation on how to perform the yoke walk based on the technique used by experienced strongman athletes; conceptualise technique improvements for performance enhancement; identify possible injury risks associated with performing the yoke walk; prescribe the use of the yoke walk as a training tool for both strongman and non-strongman athletes; and direct future research into the strongman yoke walk.

## Materials and Methods

### Experimental Approach

A cross-sectional observational experimental design was used to establish spatiotemporal and kinematic biomechanical characteristics throughout a 20 m yoke walk. Male and female strongman athletes (Table 1) undertook two testing sessions. Session one consisted of a determination of the athlete's 20 m yoke walk one-repetition-maximum (1RM) to establish loading conditions for session two. Session two consisted of the collection of spatiotemporal and kinematic measures during three sets of 20 m yoke walks with 85% 1RM load. Anthropometric measures of stature, body mass, trochanterion-tibiale laterale height and tibiale laterale height of each athlete were taken by a trained person using ISAK methodologies (Marfell-Jones et al., 2012).

**Table 1:** Participant characteristics.

**Descriptor**	**Female**	**Male**
Age (y)	33.1 ± 6.7	30.3 ± 6.8
Body mass (kg)	81.1 ± 14.5	111.5 ± 26.8
Stature (m)	1.65 ± 0.04	1.82 ± 0.09
Femur length (m)	0.394 ± 0.032	0.420 ± 0.040
Tibia length (m)	0.475 ± 0.022	0.519 ± 0.030
Max 20 m yoke (kg)	170.0 ± 44.0	270.0 ± 41.6
85% 1RM yoke (kg)	144.5 ± 37.4	229.5 ± 35.3
Strongman training experience (years)	2.5 ± 1.0	2.9 ± 1.7
Strongman competition experience (number of competitions in past 2 years)	4.0 ± 3.0	3.4 ± 2.2

### Participants

Nineteen experienced strongman competitors (12 male and 7 female) were recruited for this study (Table 1). All participants were required to have a minimum of 18 months' strongman training experience, have competed in at least one strongman competition and be free from moderate or major injury for a minimum of 1 week before testing. For the purposes of the study a moderate injury was defined as an injury that had stopped the athlete from performing a strongman exercise during a strongman session, whereas a major injury was defined as an injury which had stopped the athlete continuing all exercises/the session completely (Keogh and Winwood, 2017). Participants who met the above criteria were informed of the purpose of the study and asked to sign an informed consent form. Ethical approval was granted for all procedures used throughout this study by Bond University's Human Research Ethics Committee (BH00045).

### Trial Conditions

Athletes were instructed to prepare for each session in the same way in which they would prepare for a training session to achieve optimal performance in the testing sessions. As athletes were well-trained in strongman, self-directed warm up routines were performed by each participant (Winwood et al., 2014a, 2015a,b, 2019; Renals et al., 2018). Warm up routines typically lasted for 15–30 min and included dynamic stretching and short distance (<10 m) yoke walks at loads approaching those expected to be used by the individual throughout the session. Athletes were permitted to use knee and elbow sleeves, lifting belts, wrist wraps and lifting chalk during sessions, as these lifting aids are commonly used in training and competition.

### Session Protocols

Session one 1RM testing required athletes to carry a maximal load yoke a distance of 20 m in under 20 s without dropping (returning the yoke to the ground) during the walk. Athletes worked up to a maximum yoke load in increments selected by the athlete. When an athlete was unable to complete the distance in under 20 s, or dropped the yoke before finishing the 20 m, the athlete was permitted one additional attempt at the failed load. Where the athlete failed the second attempt, the previous successfully completed load was prescribed as their 1RM. Athletes were assigned a rest period of six to eight min between each attempted load (Winwood et al., 2011).

Session two was performed a minimum of seven days after session one and required athletes to perform three sets of a 20 m yoke walk as quickly as possible at a load of 85% of their 1RM from session one. This load was selected to reflect a typical training session routinely performed by the strongman athletes, whereby they typically select heavy, submaximal loads with the intention of performing multiple sets with high velocity and no drops. To begin the trial the athlete was positioned standing beneath the cross member of the yoke with the yoke still in contact with the ground, as would be the typical starting position in a strongman competition. On the signal “athlete ready, three, two, one, lift” the athlete lifted the yoke from the ground and commenced the 20 m walk. The trial was concluded as soon as the final timing gate was broken at the 20 m line. Where an athlete dropped the yoke during a set, data were only included from the previously completed 5 m intervals within that set. Athletes were assigned a rest period of six to eight min between each set (Winwood et al., 2011).

### Data Acquisition and Analysis

**Figure 2:**
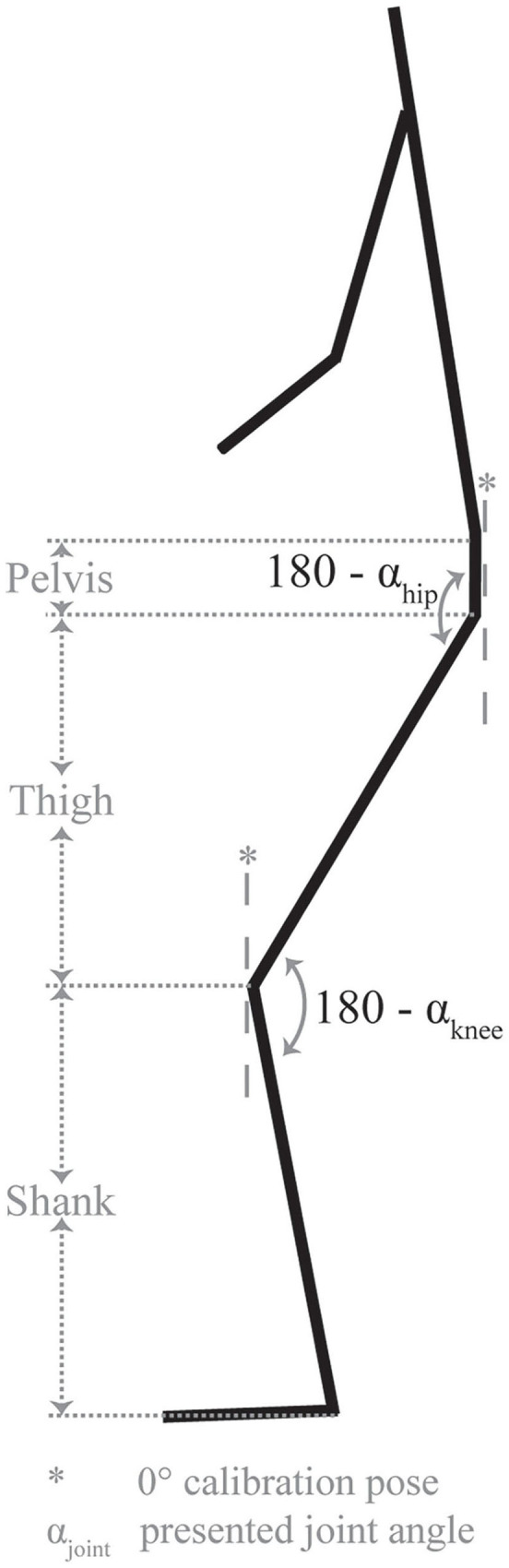
Joint angle definitions.

Yoke walks were performed indoors on a 20 m rubberised/synthetic floored runway. Dimensions of the yoke were 1.58 m (length), 1.38 m (width), 2.08 m (height), with an adjustable crossmember to suit the stature of each athlete. Kinematic and spatiotemporal measures of athletes performing the yoke walk were estimated using the inertial-based motion capture methodologies of Hindle et al. (2020) (Table 2). Four magnetic angular rate and gravity (MARG) devices (ImeasureU, Vicon Motion Systems Ltd., Oxford, UK) were positioned on the athlete as detailed in Table 3, capturing tri-axial acceleration, angular velocity and magnetic field strength data at 1125 Hz (accelerometer and gyroscope) and 112 Hz (magnetometer) (Hindle et al., 2020). The MARG data collected for each segment were input into a Matlab script (The Mathworks Inc., Natick, MA, USA) developed by the authors to estimate hip and knee joint kinematics in the sagittal plane (Figure 2), and stride length, stride rate and stance duration (Hindle et al., 2020).

**Table 2:** Temporal and kinematic measurement definitions.

**Parameter**	**Definition**
Spatiotemporal	
Mean velocity (m/s)	Distance of the walk interval (5 m) divided by the time taken to complete the given interval.
Stride rate (Hz)	Inverse of the time for each stride.
Stride length (m)	Horizontal distance covered from heel strike to the next heel strike of the same foot.
Stance duration (s)	Duration of time from heel strike to toe-off of the same foot.
Kinematic	
Joint angle (°)	Hip and knee angle at heel strike and toe-off. Joint angle definitions provided in Figure 3. Positive angles denote flexion, negative angles denote extension.
Hip ROM (°)	Maximum angle between the pelvis and thigh minus minimum angle between the pelvis and thigh throughout a stride.
Knee ROM (°)	Maximum angle between the thigh and shank minus minimum angle between the thigh and shank throughout a stride.

*ROM, range of motion*.

**Table 3:** MARG device locations.

**Segment**	**Position**
Pelvis	Halfway between the left and right posterior superior iliac spine
Right thigh	150 mm proximal to the lateral epicondyle of the femur
Right shank	100 mm distal to the lateral tibial condyle
Right foot	Midway between the base of the foot and the lateral malleoli

**Figure 3:**
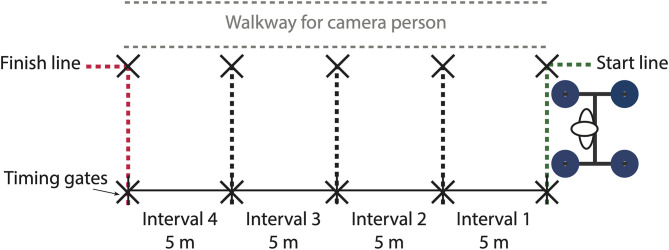
Runway and equipment schematic.

Timing gates (Smartspeed, Fusion Sport, Queensland, Australia) were positioned at the 0 m (start), 5, 10, 15, and 20 m (finish) mark of the runway (Figure 3) to measure split times for each 5 m interval. All velocity measures reported throughout were based on timing gate calculations. At the beginning of each trial the yoke was positioned behind the 0 m mark so that the first timing gate would be broken within the first stride made by the athlete. An iPad Air 2 (iPad Air 2, iOS 13.3.1, Apple Inc., CA, USA) recording at 120 Hz was used to capture and count complete strides within each 5 m interval. The video data were used to identify strides from each interval in the time-series MARG-based spatiotemporal and kinematic estimations (Table 2). Spatiotemporal measures of mean velocity, stride length, stride rate and stance duration were normalised using a Froude number approach to account for between-athlete (especially, between-sex) differences in lower limb length and inertial properties (Hof, 1996; Bruening et al., 2020). Only normalised spatiotemporal measures were included in the statistical analysis. Non-normalised values are provided in the Supplementary Tables.

### Statistical Methods

Descriptive statistics (mean ± standard deviation) of all variables were calculated for each 5 m interval of the 20 m walk. A linear mixed effects model with *post-hoc* analyses was used to establish two-way interactions between sex and interval for each biomechanical measure and main effects of sex, interval and set. Each individual athlete was classified as a random effect. The modelled data was assessed for main effects of set prior to combining measured parameters for all sets. Partial eta-squared effect sizes ($\eta_{\text{p}}^{2}$) were calculated for two-way interactions with classifications of negligible ($\eta_{\text{p}}^{2}$ ≤ 0.01), small (0.01 > $\eta_{\text{p}}^{2}$ ≥ 0.06), moderate (0.06 > $\eta_{\text{p}}^{2}$ ≥ 0.14) and large ($\eta_{\text{p}}^{2}$ > 0.14) (Cohen, 1988). Bonferroni *post-hoc* pairwise *t*-tests were conducted on parameters where significant differences were detected. Cohen's d (d) effect sizes were calculated for pairwise comparisons with classification of negligible (*d* < 0.2), small (0.2 ≤ *d* < 0.5), moderate (0.5 ≤ *d* < 0.8) and large (*d* ≥ 0.8) (Cohen, 1988). Data were checked for normality and homoscedasticity using visual inspection. Power analyses were conducted based on the limited farmers walk data available (Keogh et al., 2014). Expected between-interval differences indicated a total population of 17 athletes would be required to attain a study of 80% power with a Type I error of <5%. Based on previous between-sex data of load carriage (Silder et al., 2013; Bode et al., 2021), significant, albeit small and in some cases no between-sex differences were reported. Using the data of Bode et al. (2021), in which significant between-sex differences in knee joint ROM were observed during load carriage (≤55 kg), a sample size of ~16 male and 16 female strongman athletes would be required to attain a study of 80% power with a Type I error of <5%. All statistical analyses were performed in R version 3.6.1 (R Development Core Team, Vienna, Austria), with statistical significance accepted at *p* = 0.05.

## Results

A total of 854 strides were collected across all participants and trials, providing data of 854 and 839 strides for the hip and knee, respectively. The failure to analyse knee joint kinematics for all strides was attributed to sensor malfunction (*n* = 15). Spatiotemporal measures were collected for all 854 complete strides. Data were omitted from interval two (*n* = 1), interval three (*n* = 1) and interval four (*n* = 2), where participants (*n* = 2) dropped the yoke during a set. Anthropometric measures of stature (*d* = 2.59, *p* < 0.001), body mass (*d* = 1.41, *p* = 0.005) and lower limb length (*d* = 1.35, *p* = 0.009) statistically differed between male and female athletes, therefore all relevant variables were normalised to remove lower limb anthropometric effects.

### General Biomechanical Characterisation

**Figure 4:**
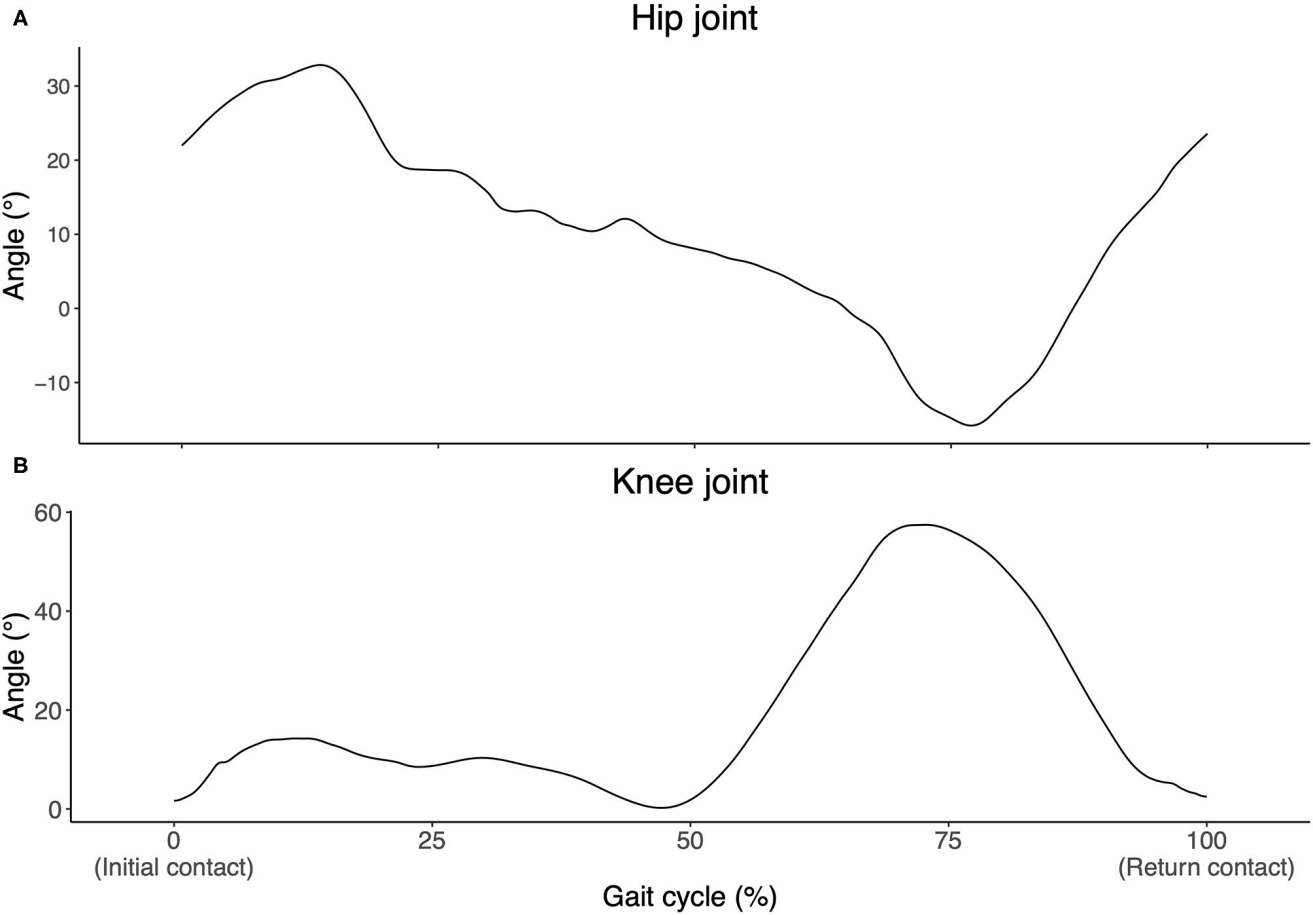
Hip **(A)** and knee **(B)** joint angle exemplar data for a single gait cycle of the yoke walk. Flexion denoted by a positive angle and extension denoted by a negative angle.

Mean and standard deviation of the kinematic and spatiotemporal measures for the entire yoke walk are presented in Table 4. Notable kinematic characteristics of the yoke walk included: flexion of the hip and slight to neutral flexion of the knee at heel strike, slight to neutral extension of the hip and flexion of the knee at toe-off (Figure 4, Supplementary Table 1). Statistically significant differences in hip and knee joint angles between heel strike and toe off events were supported by large effect sizes (hip: *d* = 3.53, *p* < 0.001; knee: *d* = 5.08, *p* < 0.001) (Supplementary Table 2). No statistically significant main effect between-sex differences were observed for both kinematic and spatiotemporal measures (0.004 ≤ $\eta_{\text{p}}^{2}$ ≤ 0.118, *p* ≥ 0.15) (Table 4, Supplementary Table 4).

**Table 4:** Spatiotemporal and kinematic interval independent mean ± SD measures of the yoke walk.

	**Male**	**Female**	**Group**
**Spatiotemporal**			
Mean velocity (m/s)	1.649 ± 0.367	1.770 ± 0.326	1.694 ± 0.356
Normalised mean velocity	0.546 ± 0.121	0.604 ± 0.111	0.567 ± 0.121
Stride length (m)	1.127 ± 0.174	1.155 ± 0.164	1.138 ± 0.171
Normalised stride length	1.211 ± 0.187	1.320 ± 0.188	1.252 ± 0.194
Stance duration (s)	0.435 ± 0.061	0.389 ± 0.040	0.417 ± 0.058
Normalised stance duration	1.410 ± 0.198	1.303 ± 0.135	1.370 ± 0.184
Stride rate (Hz)	1.586 ± 0.201	1.670 ± 0.123	1.617 ± 0.180
Normalised stride rate	0.489 ± 0.062	0.499 ± 0.037	0.492 ± 0.054
**Hip**			
Initial contact (°)	23.4 ± 6.8	24.3 ± 7.5	23.8 ± 7.1
Toe-off (°)	−2.9 ± 7.5	−3.8 ± 9.2	−3.2 ± 8.2
Range of motion (°)	36.5 ± 7.7	40.4 ± 7.3	37.9 ± 7.8
**Knee**			
Initial contact (°)	6.7 ± 4.8	4.0 ± 4.0	5.7 ± 4.7
Toe-off (°)	46.2 ± 8.6	45.4 ± 12.3	45.9 ± 10.2
Range of motion (°)	54.6 ± 10.0	52.7 ± 11.7	53.9 ± 10.7

### Between-Interval Biomechanical Differences–Sex Independent (Main Effect)

**Figure 5:**
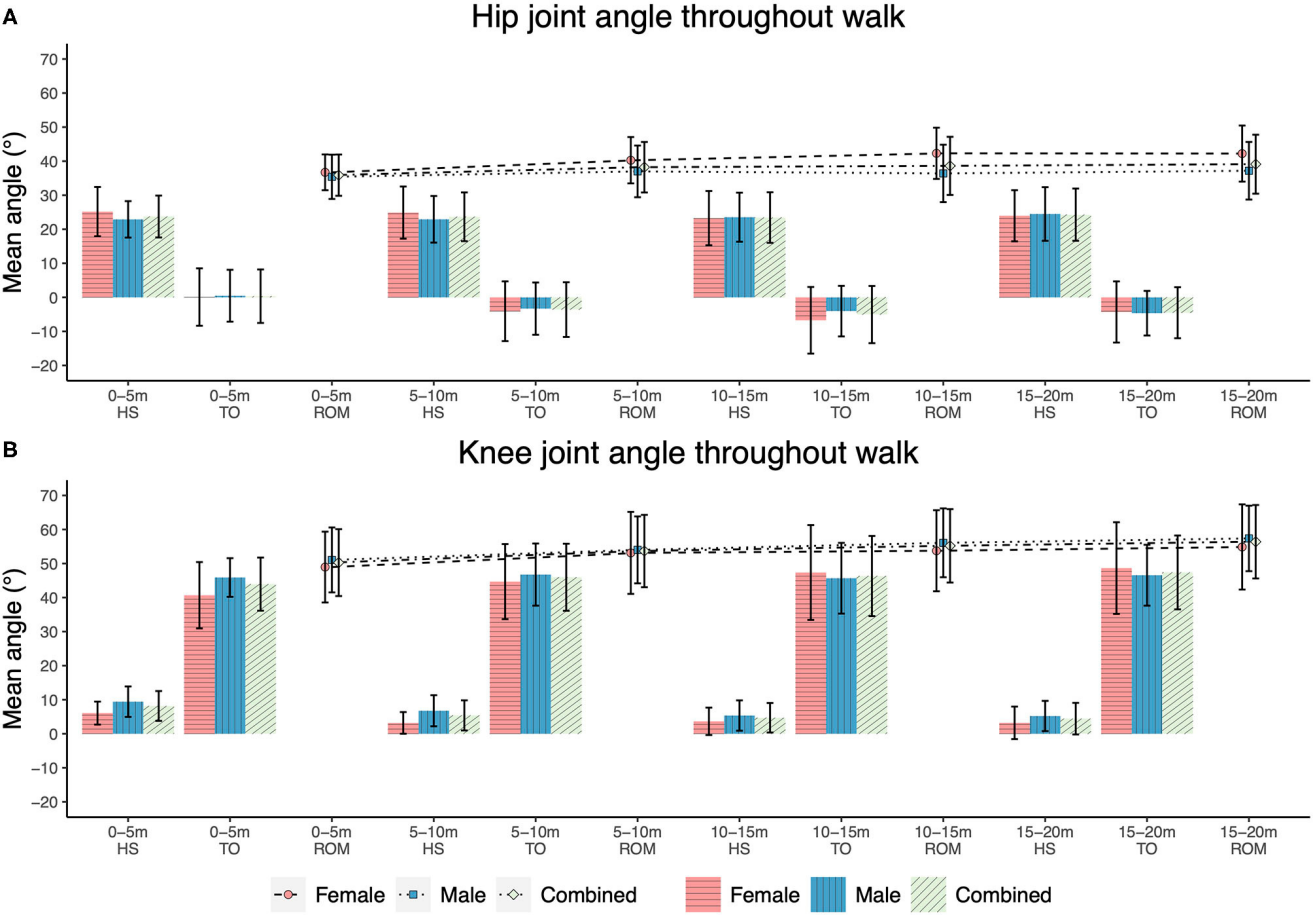
Joint ROM kinematic measures for each 5 m interval of the 20 m yoke walk. **(A)** hip joint kinematics; **(B)** knee joint kinematics.

**Figure 6:**
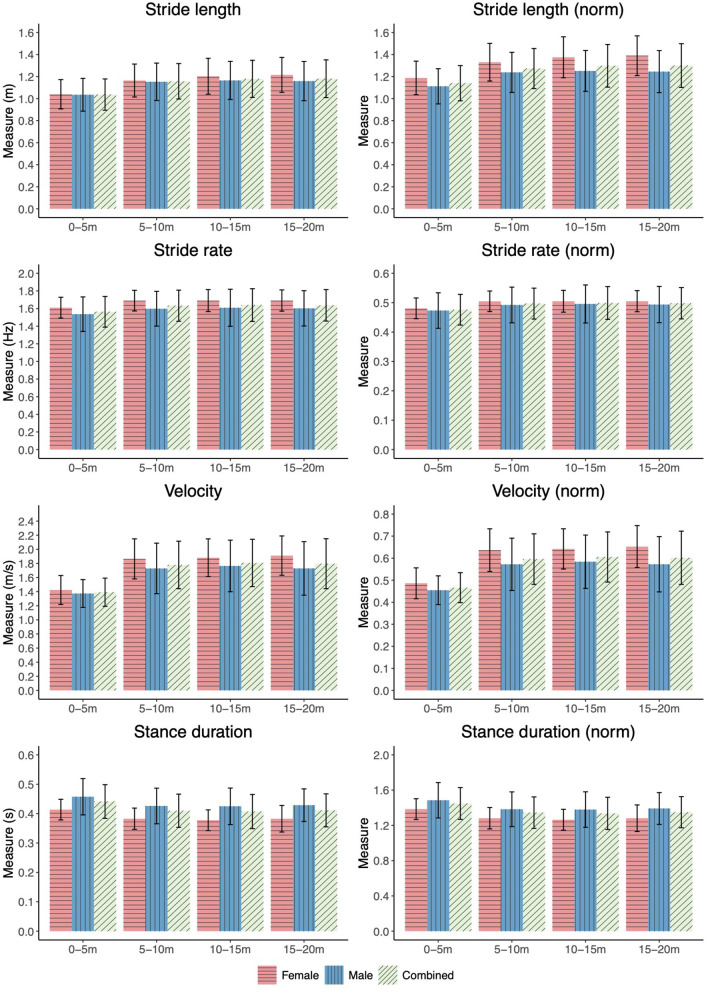
Spatiotemporal measures for each 5 m interval of the 20 m yoke walk.

A number of statistical between-interval joint kinematic and spatiotemporal differences were observed (Figure 5 and Supplementary Table 1). Small to large effect sizes were presented for knee joint angle at heel strike (0.69 ≤*d* ≤ 0.96, *p* < 0.001), hip joint angle at toe-off (0.69 ≤ *d* ≤ 0.93, *p* ≤ 0.001) and knee ROM (−0.64 ≤ d ≤ −0.36, *p* < 0.001) between combinations of the first interval and later three intervals (Supplementary Table 3). For normalised spatiotemporal parameters, athletes exhibited statistically smaller stride length (−0.97 ≤*d* ≤ −0.80, *p* < 0.001), stride rate (−0.47 ≤*d* ≤ −0.43, *p* < 0.001) and mean velocity (−1.53 ≤*d* ≤ −1.42, *p* < 0.001), and increased stance duration (0.60 ≤ *d* ≤ 0.69, *p* < 0.001) in the initial interval when compared with the final three intervals (Figure 6), with effect sizes generally ranging from small to large (Supplementary Tables 1, 3).

### Between-Interval Biomechanical Differences–Sex Dependent (Two-Way Interaction)

Small effect sizes were observed for two-way interactions between sex and interval for measures of knee angle at toe-off ($\eta_{\text{p}}^{2}$ = 0.048, *p* = 0.022), hip ROM ($\eta_{\text{p}}^{2}$ = 0.048, *p* = 0.020) and normalised stride length ($\eta_{\text{p}}^{2}$ = 0.040, *p* = 0.045) (Supplementary Table 4). Female athletes exhibited significantly greater knee extension at toe off during the initial interval when compared with the final two intervals (0.66 ≤*d* ≤ 0.79, 0.001 ≤*p* ≤ 0.008) and reduced hip ROM during the initial interval when compared with the final three intervals (−0.97 ≤ d ≤ −0.62, 0.001 < *p* ≤ 0.006), whereas male athletes did not display these between-interval differences (Supplementary Tables 1, 3, and Figure 5). In addition to the statistically smaller normalised stride length observed during the initial interval when compared with the final three intervals observed for male and female athletes, female athletes also displayed smaller normalised stride length during interval two when compared with interval four (*d* = −0.373, *p* = 0.033) (Figure 6).

### Between-Set Biomechanical Differences

Between-set analysis was performed for the purpose of identifying any potential effects of set number (possibly indicative of fatigue) on athlete biomechanics. A number of statistical between-set biomechanical differences were observed for the combined group (Supplementary Table 6). Pairwise comparisons revealed between-set differences to be primarily between sets one and three, with all differences being of a negligible to small effect size (−0.47 ≤*d* ≤ 0.16, 0.001 ≤ *p* ≤ 0.04) (Supplementary Tables 7, 8). Due to the negligible to small effect sizes observed for between-set differences, data from each set were combined for all analyses.

## Discussion

The aim of this study was to use ecologically realistic training loads and carry distances to (1) establish the preliminary biomechanical characteristics of the yoke walk; (2) identify any biomechanical differences between male and female athletes performing the yoke walk; and (3) determine spatiotemporal and kinematic differences between stages (intervals) of the yoke walk. The research provides an initial description of the observed spatiotemporal and kinematic characteristics of experienced strongman athletes carrying loads similar to those seen in competition.

### General Biomechanical Characterisation–Sex Independent

Throughout the gait cycle of the yolk walk, athletes presented flexion of the hip and slight to neutral flexion of the knee at heel strike, slight to neutral extension of the hip and flexion of the knee at toe-off and moderate hip and knee ROM. When compared with the previously studied farmers walk, athletes exhibited reduced flexion of the knee at heel strike (farmers walk: 25.0 ± 7.3°; yoke walk: 5.7 ± 4.7°) and toe-off (farmers walk: 54.4 ± 8.7°; yoke walk: 45.9 ± 10.2°), and greater knee ROM (farmers walk: 29.0 ± 11.6°; yoke walk 53.9 ± 10.7°) during the yoke walk (Keogh et al., 2014). Shorter stride length (farmers walk: 1.54 ± 0.13 m; yoke walk: 1.14 ± 0.17 m), lower stride rate (farmers walk: 1.89 ± 0.13 Hz; yoke walk: 1.62 ± 0.18 Hz) and increased stance duration (farmers walk: 0.32 ± 0.04 s; yoke walk: 0.42 ± 0.06 s) were also reported for the yoke walk when compared with the farmers walk (Keogh et al., 2014), with such differences likely due to the higher loads used in the yoke walk (yoke walk: 198.2 ± 54.8 kg; farmers walk: 181 ± 0.0 kg).

As described by Hindle et al. (2019), a physical limit exists where the load carried becomes so great that the athlete is not able to continue to increase or maintain their stride rate to compensate for the decrease in stride length, and thus a decrease in velocity occurs. The lower stride rate and stride length reported for the yoke walk when compared with the farmers walk (Keogh et al., 2014) further highlights the inability of the athlete to continue to increase their stride rate to compensate for the loss of stride length under heavier loading. Identifying the threshold load or %1RM where stride rate begins to decrease may be of interest to strongman coaches and strength and conditioning coaches using loaded walks to target foot speed, core stability and total body strength adaptations (Winwood et al., 2014b).

Data from the current study indicates that from heel strike until the end of the double support phase, the knee is in a mostly extended state. The combination of an extended knee throughout the stance phase and a short stride length reduces the vertical displacement of the athlete's centre of mass (COM) (Kuo, 2007), reducing the chance of “catching” the yoke on the ground. Where a reduced stride length requires an increase in stride rate to achieve an equivalent velocity, an increase in metabolic demand is expected (Gordon et al., 2009; Lieberman et al., 2015). The increase in metabolic demand may however, be overcome by the reduced energy expenditure caused by; the reduced moments around the knee (as a result of reduced knee flexion during stance) (Alexander, 1991), and the reduced requirement to lift the total system load against gravity (as a result of the reduced vertical COM displacement). When compared with the farmers walk, athletes performing the yoke walk exhibited both greater extension of the knee at heel strike and shorter stride lengths (Keogh et al., 2014). A reduced vertical COM displacement may be particularly important when performing the yoke walk due to the naturally smaller ground-to-implement clearance and greater total system load being carried when compared with the farmers walk. While the reduction in stride length supports hypothesis one, the increase in lower limb (knee) ROM and stance duration, and reduction in stride rate, as a result of the load threshold appearing to be crossed, is contrary to what was initially hypothesised.

### General Biomechanical Characterisation–Sex Dependent

No differences were observed for the general biomechanical characteristics of the yoke walk between male and female athletes. Although conclusions of previous between-sex load carriage biomechanical studies are varied, the findings of the current study are in line with the lack of between-sex sagittal plane kinematic and spatiotemporal differences observed in previous literature using body-mass relative loading (≤30% body mass) (Silder et al., 2013) and relatively light absolute loads (i.e., 22 kg) (Krupenevich et al., 2015). The vastly different absolute loads and study populations in Silder et al. (2013) and Krupenevich et al. (2015) compared to the current study, should however, be acknowledged.

While the current study only assessed kinematics in the sagittal plane, potential sex-related differences in frontal and transverse plane kinematics, muscle activation and anthropometrics have been hypothesised as rationale for the greater occurrence of anterior cruciate ligament and patellofemoral injuries reported in females (Malinzak et al., 2001; Ferber et al., 2003; Ford et al., 2003). Further investigation into transverse and frontal plane kinematics and muscle activation patterns of male and female strongman athletes performing the yoke walk is expected to assist in identifying any sex-specific injury risks associated with the yoke walk exercise. The lack of spatiotemporal and sagittal plane kinematic between-sex differences observed in the current study supports hypothesis two.

### Between-Interval Biomechanical Differences–Sex Independent (Main Effect)

Athletes exhibited shorter stride length, increased stance duration, reduced stride rate and mean velocity, greater knee flexion at heel strike and hip flexion at toe off and reduced knee ROM during the initial interval (0–5 m) of the yoke walk when compared with the final three intervals (5–20 m). Such observations were consistent with differences between the initial and later intervals of a 20 m farmers walk (Keogh et al., 2014). The greater knee flexion at heel strike and hip flexion at toe off observed in the initial interval of the yoke walk likely contribute to the smaller knee ROM and stride length observed in the initial interval when compared with the later intervals. The abbreviated knee ROM may be a mechanism employed by athletes to rapidly increase stride rate during acceleration before achieving maximal velocity as soon as possible, through the optimisation of stride length (as a result of increased lower limb ROM), in the later intervals (Murphy et al., 2003).

Although kinetic outcomes were not directly measured in the current study, the statistically greater change in velocity between interval one and interval two of the yoke walk when compared with all other immediately successive intervals, suggest a greater horizontal impulse applied by the athlete during the first interval (acceleration phase). This can be deduced in accordance with the impulse-momentum relationship and is supported by previous research on impulse differences between acceleration and maximal velocity phase sprinting (Nagahara et al., 2018). Ballistic training may be of benefit to athletes when preparing for a yoke walk competition event in order to develop the neuromuscular capacities required to generate maximal force and thus greater propulsive impulse during relatively short periods of ground contact (Cormie et al., 2011; Samozino et al., 2012). The observed differences in spatiotemporal and kinematic parameters between the initial acceleration (0–5 m) and later maximal velocity (5–20 m) intervals are in support of hypothesis three of the study.

### Between-Interval Biomechanical Differences–Sex Dependent (Two-Way Interaction)

Both male and female athletes exhibited shorter normalised stride length during the initial interval when compared with the final three intervals. Female athletes, however, also exhibited statistically shorter normalised stride length during the second interval than the final interval, indicating female athletes cover a greater distance before reaching maximal stride length than male athletes. Although female athletes in the current study had statistically shorter lower limb lengths than males, stride length was normalised to lower limb length, eliminating the effect of lower limb anthropometry on stride length. The observed difference, may however, be the result of males having a greater prevalence of type II fibres than females (Haizlip et al., 2015). The greater prevalence of type II muscle fibres gives male athletes an advantage over female athletes during the acceleration phase where rapid force production is key to achieving maximal stride rate and stride length as quickly as possible.

Female athletes during the initial interval displayed greater extension of the knee at toe-off when compared with the final two intervals, and smaller hip ROM when compared with the final three intervals, whereas male athletes did not exhibit these characteristics. Similar to the suggested mechanism of reducing knee ROM to increase stride rate, as was observed for the group dataset, the greater extension of the knee at toe off and reduced hip ROM during the first interval when compared with the final intervals may have been a further mechanism employed by female athletes to increase stride rate and overcome the inertia of the load during the initial 5 m of the walk. Where the main effects of sex on the biomechanics of athletes performing the yoke walk supported hypothesis two, in that, no between-sex differences were observed, the identified two-way interactions between sex and interval provide means to reject this hypothesis.

### Additional Considerations

The current study has a number of limitations which should be addressed. The IMU-based methodology used in the study has been reported to produce joint kinematic estimations which are highly representative of those estimated using an optical motion capture system for such functional fitness exercises as the squat (hip MAPE: 8.2 ± 6.5%; knee MAPE: 5.1 ± 3.7%), box squat (hip MAPE: 6.8 ± 6.1%; knee MAPE: 4.0 ± 2.7%) and sandbag pickup (hip MAPE: 7.0 ± 5.5%; knee MAPE: 3.7 ± 2.8%) (Hindle et al., 2020). This methodology, however, showed less agreement for knee (MAPE: 22.5 ± 16.5 %) and hip (MAPE: 25.1 ± 21.0%) joint kinematics during a small ROM (hip: 14.3 ± 3.7°; knee: 22.9 ± 8.0°) shuffle gait pattern (Hindle et al., 2020). Although care should be taken when interpreting joint kinematic results in this study, greater validity may be expected for hip and knee joint kinematics of athletes performing the yoke walk than a shuffle walk gait pattern due to a reduced number of dynamic degrees of freedom caused by the increase in the complexity of the task (loading) (Jordan et al., 2006; Barrett et al., 2008; Poitras et al., 2019) and the greater ROM observed during the yoke walk (hip: 37.9 ± 7.8°; knee: 53.9 ± 10.7°). Where ankle joint ROM in previous load carriage exercises have been reported to be small [Winwood et al. (2014a): 9.6 ± 9.8°; Keogh et al. (2014): −3.0 ± 4.0°], the IMU-based methodology used was declared to be inappropriate for this application due to the expected small ROM of the ankle during the yoke walk, thus ankle joint measures were not included in this study.

The number of male (*n* = 12) and female (*n* = 7) athletes included in the current study are individually larger than the majority of previous strongman biomechanics studies (McGill et al., 2009; Keogh et al., 2010a,b; Keogh et al., 2014; Winwood et al., 2014a, 2015a,b). Nevertheless, the power analysis performed and relatively large between-sex effect sizes, large confidence intervals and corresponding statistical insignificance (*p* > 0.05) observed in some measurement parameters, indicates an under-powered sample size for between-sex comparisons (Fritz et al., 2012). Where possible, future studies should look to include a greater number of male and female athletes of similar competitive standard for between-sex analyses.

As this is the first study to measure kinematic and spatiotemporal parameters of the yoke walk there is significant scope for future research, including: transverse and frontal plane kinematic analyses; establishing relationships between anthropometrics and biomechanical characteristics of athletes; the effect of yoke load on the biomechanics of an athlete; and the biomechanical determinants of greater performance in the yoke walk. Such research is expected to equip strongman athletes and coaches, and strength and conditioning coaches with the knowledge required to elicit greater performance when undertaking the yoke walk or similar heavy load carriage exercises whilst minimising the risk of injury to the athlete.

## Conclusion

This study provides the first descriptive data of the spatiotemporal and joint kinematic characteristics of male and female strongman athletes performing the yoke walk. The yoke walk presented a number of differences in spatiotemporal and kinematic measures when compared with previous load carriage research of the farmers walk and backpack load. Between-interval spatiotemporal and joint kinematic differences were observed between the initial (lower velocity/acceleration) and later (maximal velocity) intervals. No main between-sex differences and a limited number of two-way interactions between sex and interval were observed. It is suggested that an abbreviated lower limb ROM during the initial intervals will assist in rapidly increasing stride rate, and therefore velocity. Further, the combination of a short stride length and high stride rate is suggested to minimise vertical yoke displacement and metabolic demand placed on the athlete while performing the yoke walk. The results of this biomechanical analysis of the yoke walk provides a preliminary description of the movement that will: assist strongman training and competition performance, improve strength and conditioning coaching practise for coaches interested in prescribing this exercise to non-strongman athletes and establish significant scope for future research.

## Data Availability Statement

The datasets generated for this study can be found in online repositories. The names of the repository/repositories and accession number(s) can be found below: https://cloudstor.aarnet.edu.au/plus/s/QpOZCGtN4RB5DhZ.

## Ethics Statement

The studies involving human participants were reviewed and approved by Bond University Human Research Ethics Committee. The patients/participants provided their written informed consent to participate in this study. Written informed consent was obtained from the individual(s) for the publication of any potentially identifiable images or data included in this article.

## Author Contributions

BH assisted in devising the study protocol, collected data, processed and analysed the data, performed statistical analysis, compiled the manuscript. AL, PW, and JK assisted in devising the study protocol, assisted in analysing the data, drafted, and wrote sections of the manuscript. DB assisted in devising the study protocol, collected data, assisted in analysing the data, drafted, and wrote sections of the manuscript. All authors contributed to the article and approved the submitted version.

## Conflict of Interest

The authors declare that the research was conducted in the absence of any commercial or financial relationships that could be construed as a potential conflict of interest. Dr. Justin Keogh who was one of the editors of this special issue.

## Supplementary Material

Numerical data supporting Figures 5, 6 (including effect sizes) are provided in spreadsheet form as Supplementary Material (Supplementary Tables 1–8).
